# Adult-Centred Systems, Youth-Centred Needs: A Qualitative Study of Canadian Caregiving Service Providers’ Readiness to Support Young Caregivers

**DOI:** 10.3390/ijerph23020180

**Published:** 2026-01-31

**Authors:** Kristine Newman, Luxmhina Luxmykanthan, Arthur Ze Yu Wang, Heather Chalmers

**Affiliations:** 1 Daphne Cockwell School of Nursing, Toronto Metropolitan University, 288 Church Street, Toronto, ON M5B 1Z5, Canada; luxmhina@torontomu.ca (L.L.); ze.wang@torontomu.ca (A.Z.Y.W.); 2 Child and Youth Studies, Brock University, 1812 Sir Isaac Brock Way, Saint Catharines, ON L2S 3A1, Canada; hchalmers@brocku.ca

**Keywords:** young caregivers, service providers, caregiving organizations, qualitative, Canada

## Abstract

**Highlights:**

**Public health relevance—How does this work relate to a public health issue?**

- Young caregivers are a large but underrecognized population whose unpaid care sustains families and health systems, yet they remain largely invisible within Canada’s adult-centred caregiving infrastructure.
- Caregiving organizations play a critical public health role in identifying, supporting, and mitigating risks for young caregivers, but their capacity to do so remains uneven and constrained.

**Public health significance—Why is this work of significance to public health?**

- This study provides rare multi-provincial insight into caregiving organizations’ readiness to support young caregivers, shifting the focus from individual burden to community and system-level capacity and gaps.
- Findings demonstrate how policy invisibility, funding instability, and adult-oriented service models limit preventative and early-intervention approaches for young caregivers.

**Public health implications—What are the key implications or messages for practitioners, policy makers and/or researchers in public health?**

- Public health policy must formally recognize young caregivers and invest in youth-centred, preventative service models integrated across health, education, and community sectors.
- Practitioners and researchers should prioritize early identification pathways, school-based outreach, and scalable virtual supports to reduce reliance on crisis-driven interventions.

**Abstract:**

Young caregivers, defined as individuals under 25 years of age who provide unpaid care to a family member(s) with illness, disability, or age-related needs, remain significantly underrecognized in Canada despite their valuable contributions to the healthcare system. Limited awareness, fragmented services, and adult-centred caregiving infrastructures leave them vulnerable to social isolation, disrupted education, and poor mental health. Unlike the United Kingdom and Australia, Canada lacks a coordinated national strategy to identify and support young caregivers. This qualitative study examines caregiving organizations across multiple Canadian provinces, exploring current practices, barriers, and future visions for supporting young caregivers. Group interviews were conducted with 18 service providers from caregiving organizations in Alberta, BC and Nova Scotia. Four themes emerged through analysis: (1) The Landscape of Existing Caregiving Organizations, (2) Barriers and Challenges to Supporting Young Caregivers, (3) Navigating a Pandemic, and (4) a Journey and Vision Worth Supporting. Organizations reported a strong interest in expanding support for young caregivers with a vision for cross-sector collaboration and school-based outreach. However, challenges such as inadequate funding and a lack of formal recognition limits their capacity in building youth programs. Findings from the study highlight the need for systemic reform, including early intervention models, sustainable funding, and formal recognition of young caregivers within policy frameworks. Addressing these gaps will not only uplift young caregivers, but also strengthen Canada’s broader caregiving and healthcare ecosystem.

## 1. Introduction

Young caregivers (also known as young “carers” outside of North America) are an underrecognized population, defined as individuals under the age of 25 who provide unpaid care to family members living with mental and physical challenges that impact their ability to perform activities of daily living [1,2,3]. In 2018, about 1.5 million Canadian youth aged 15 to 30 reported providing care or help to a family member or friend with a long-term condition, physical or mental disability, or problems related to aging [4]. Each young caregiver’s unpaid labour is estimated to save the Canadian healthcare system and their family between $25,000 and $50,000 every year [3] and more recent studies approximating family caregivers contributing an annual value of $97.1 billion in unpaid care work [5].

Despite the value of their contributions, young caregivers experience substantially poorer mental health outcomes than their non-caregiving peers, including heightened risks of social isolation, psychological distress, depression, and suicidal ideation [3,6,7,8]. The demands of caregiving responsibilities can place young caregivers at the risk of becoming overburdened, contributing to negative outcomes across school, employment, health, and well-being.

According to a systematic review on health outcomes and psychosocial determinants in young carers, these adverse effects are particularly pronounced among young caregivers with high caregiving intensity, limited social support, and prolonged caregiving responsibilities, raising concerns about long-term and potentially lifelong consequences for the wellbeing of this group [7]. These risks were further amplified during the COVID-19 pandemic, as caregiving demands increased and access to pre-pandemic informal support was disrupted, contributing to poorer mental health and wellbeing among young carers compared to their non-carer peers [9,10,11].

Compared to countries such as the United Kingdom and Australia, it is evident that Canada has vast areas for improvement. Currently, there is no national or provincial recognition of young caregivers and there are no consistent legal or policy frameworks. Consequently, there is a lack of standardized pathways to support and protect the interests and needs of young caregivers. In Canada, only a small population of young caregivers have access to formal support services. In 2015, there were only three programs; that number has only grown to approximately 12 programs as of 2025 based on information provided by community partners [2]. In contrast, the United Kingdom has prioritized research, action, and policy surrounding young caregivers and established dedicated support programs which reach approximately 1 in 14 young caregivers with efforts spanning over 40 years [12,13]. Additionally, the 2014 Care Act in the United Kingdom formally recognizes the rights of young caregivers, providing them with legal protections and access to a range of supports, including tax breaks and allowances [6]. According to Carers Trust [14], as of 2022, at least 75 organizations providing dedicated support services for young carers (ages 0–17) and 86 organizations delivering young adult carer (ages 18–24) services across the UK along with integrated support and resources across sectors.

Despite the severity of these challenges, Canadian support services for young caregivers remain sparse. Awareness of the caregiving responsibilities of youth and young adults remains limited, across the education and healthcare sector. Paired with the lack of knowledge of this group to the general public, this results in the needs of young caregivers being left unmet [1,15,16].

Limited system capacity has contributed to service programs supporting young caregivers to remain oriented toward assistance and mitigation rather than early intervention or the prevention of long-term adverse outcomes by identifying risks before they escalate [1]. Within Canada, existing initiatives tend to focus on localized or short-term supports aimed at addressing immediate caregiving burdens, rather than structural or upstream approaches that could reduce cumulative harm over their life span.

For example, the Young Caregivers Initiative: Powerhouse Project delivers individual and group programming alongside whole-family services within the Niagara, Haldimand, and Norfolk regions as well as Hamilton and Brant with support from qualified counselors and social workers. Similarly, Hospice Toronto’s *Young Carers Program* operates a small-scale model serving the Greater Toronto Area, emphasizing emotional support, peer connection, and caregiving assistance for youth while the Ontario Caregiver Organization does similar work for teens and young adults. While the programs have articulated plans for expansion and have acknowledged gaps in their service delivery models, significant structural and systemic constraints limit their ability to scale or transition toward preventative, population-level approaches. Research consistently demonstrates that these service delivery gaps and constraints on program expansion are driven by a lack of targeted and sustained funding for young caregiver-specific initiatives and the absence of formal policy frameworks recognizing young caregivers as a distinct population [2,15]. Additional barriers include limited public and institutional awareness of young caregiving, challenges in forming partnerships with schools and school boards, and the absence of standardized mechanisms for identifying young caregivers within education and health systems [2,15,17]. Collectively, these barriers impede service providers’ ability to systematically identify young caregivers, collect and utilize data to address their needs, and deliver timely, coordinated, and preventative supports, thereby reinforcing a reliance on reactive rather than preventative models of care.

Within the Canadian context, it is important to acknowledge an event with profound international impact: the COVID-19 pandemic. The pandemic intensified the challenges faced by young caregivers as their responsibilities often increased while access to essential supports and programs became limited or inaccessible due to service shutdowns, particularly in-person services [2,18]. As a result, opportunities for respite and social connection were severely reduced [1,19]. International and Canadian young caregivers experienced the loss of school or work as forms of respite, and many were unable to access formal respite services or established support structures during lockdowns, exposing the fragility of these systems [18,19]. Despite the documented impacts, research examining how Canadian caregiving organizations were affected by the pandemic, how they adapted service delivery and how they planned improvements in tailored services for young caregivers remains limited [1]. This gap highlights the need for further study of organizational responses, service resilience, and policy frameworks that can better support young caregivers in future public health emergencies.

This study addresses gaps in the existing Canadian literature by examining caregiving organizations that primarily serve adult caregivers but have expressed a desire to formally support young caregivers. It explores how existing caregiving organizations currently engage with young caregivers, the limitations and barriers they encounter, and the changes they identify as necessary to build capacity to deliver more youth-centred caregiving supports. By documenting organizational perspectives across multiple provinces, this article offers insight into the challenges facing grassroots efforts to support young caregivers and contributes to ongoing dialogue about the systemic and institutional changes required to ensure young caregivers receive appropriate, equitable, and sustainable support.

## 2. Methods

This study employed a qualitative case study methodology [20,21,22,23] to examine service providers’ experiences supporting young caregivers in Canada. Its suitability is well established when conducting small-scale research projects like ours [22] as they allow for a detailed examination of complex phenomena as they occur in real-world contexts [23], within defined temporal and spatial boundaries [21] or even gleaning insight when boundaries between phenomenon and context are not clearly delineated [24]. We thought this would be helpful given how important it is to avoid assumptions about how these organizations should and should not operate as they are only just beginning their journey to support young caregivers within diverse contexts and settings. Accordingly, a collective case study design [21] was used to provide comparisons between organizational, community, and provincial insight, contexts, and settings. While case studies often use multiple sources of data, qualitative research has shown that a single data collection method can be appropriate when it captures different perspectives within a defined setting, rather than comparing different kinds of data [25].

To provide some context on what led to this study, in our earlier work, we focused exclusively on service providers operating within Ontario and examined their efforts to support young caregivers during the COVID-19 pandemic [1]. That study highlighted both emergent practices and significant structural limitations in recognizing and supporting young caregivers within a rapidly evolving service landscape. For the study reported in this paper, we extended the scope beyond Ontario to include additional provinces where organizational infrastructure, policy recognition, and formal programming for young caregivers are less established. Expanding the geographic focus allowed us to capture a broader range of organizational contexts, service capacities and systems level challenges across Canada. Although the present study did not exclusively focus on the impacts of the COVID-19 pandemic, participants were asked to reflect on how the pandemic influenced their roles and service delivery in order to provide contextual insights on its effects.

We sampled caregiving organizations that primarily serve adults who self-identify as caregivers, and expressed a desire or emerging mandate to support young caregivers. Consistent with our prior work, the case study approach enabled an in-depth exploration of service providers’ experiences while situating their perspectives within the early stages of Canada’s efforts to recognize young caregivers as a distinct population requiring tailored supports.

Data collection was facilitated via group interviews designed to explore the following areas: (1) existing services offered to caregivers; (2) current or emerging initiatives to support young caregivers; (3) perceived challenges and barriers to extending services to this population; and (4) potential pathways for future capacity-building. Co-development of data collection instruments is a recommended practice in qualitative research to improve relevance, reflexivity, and trustworthiness of findings [26]. Accordingly, interview questions were co-developed with representatives from participating organizations to ensure contextual relevance and to enhance the depth of responses.

### 2.1. Recruitment, Setting, and Participants

Recruitment consisted of a selective sampling approach. To be included in the study, participants must have been “working in a role where you could potentially influence the lives of, potentially interact with, and/or directly interact with young caregivers and/or their families on a regular basis as part of your roles and responsibilities.” No exclusion criteria were created. Interested participants were screened via email and provided with a link to an online consent form, along with instructions for creating a participant code to support de-identification. Consent was completed and collected online via Google Forms. All interviews were conducted via Zoom by a research assistant who had over ten (10) years of field experience in qualitative data collection/analysis and seven (7) years of experience in supporting research on young caregivers. Interviews were audio-recorded and transcribed verbatim, then de-identified prior to analysis. Group interviews lasted approximately 40 min to 1 h and 30 min in duration. Data collection involved three (3) group interviews with staff and volunteers from non-profit organizations; one for each of the provinces of Alberta, British Columbia, and Nova Scotia. In total, eighteen (18) service providers participated. The service providers had one or more of the following roles: community outreach, coordinator/director, program/education development, support/resource specialist/navigator, and/or social prescribing. There were six (6) participants from Alberta, six (6) participants from British Columbia, and six (6) participants from Nova Scotia.

Group interviews followed a semi-structured format and were completed in early 2023. Although the original intention was to publish this article sooner, several factors contributed to delays, including health challenges faced by team members, and the principal investigator’s maternity leave. The team’s intentional inclusion of young caregivers as staff also introduced constraints related to time commitments and team structure. Despite the delay, the findings of this study remain relevant and there is a strong desire for these voices to be heard based on meetings and reporting from our community partners who have supported our research. This study was reviewed and received ethics clearance by Toronto Metropolitan University’s Research Ethics Board (2021-573) and Brock University (21-261).

### 2.2. Data Interpretation and Analysis

All interviews were audio-recorded and transcribed verbatim (with the exception of deidentification by the transcriber). Transcripts were analyzed using reflexive thematic analysis, an approach well suited to exploring patterns of meaning across qualitative datasets while foregrounding researcher reflexivity and positionality [27,28].

Initial familiarization and coding were conducted collaboratively by a multidisciplinary coding team consisting of: (1) a young caregiver aged 25 who began caregiving at eight years of age; (2) a PhD candidate in child and youth studies who was a Registered Psychotherapist at the time of publication; and (3) a research associate with a background in public health and law and six years of experience conducting research on young caregivers. The inclusion of coders with both lived experience and professional expertise supported a reflexive and interpretive analytic process. Coding was conducted inductively, with meaning units identified across transcripts and iteratively refined. Preliminary themes were developed through reflexive engagement with the data and were subsequently reviewed, discussed, and refined through dialogue among the three coders and the two investigators co-leading the study. This collaborative process facilitated the development of coherent primary and secondary themes that captured shared experiences across participants’ reporting while simultaneously capturing and preserving contextually nuanced and diverse narratives.

## 3. Results

**Figure 1:**
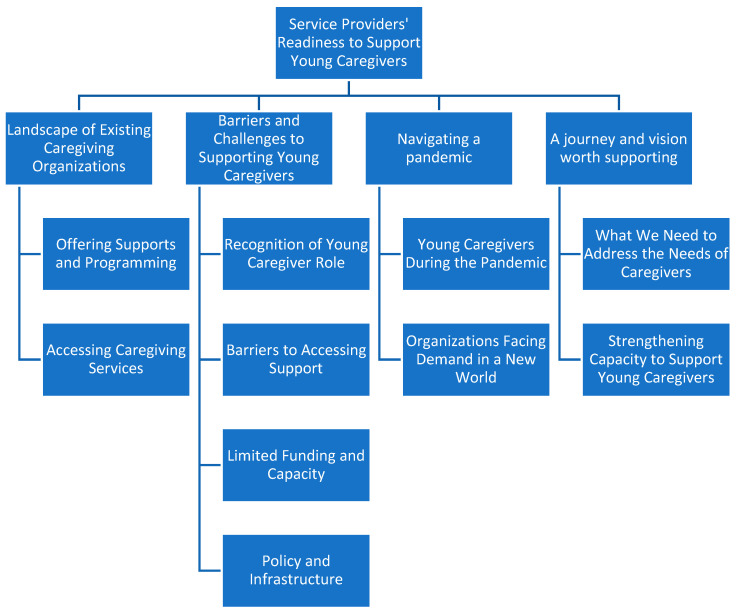
Coding tree for thematic analysis.

Four critical themes emerged from the analysis of the data: (i) Landscape of Existing Caregiving Organizations; (ii) Barriers and Challenges to Supporting Young Caregivers; (iii) Navigating a Pandemic; (iv) A Journey and Vision Worth Supporting. Figure 1 presents the themes in the form of a coding tree.

### 3.1. Landscape of Existing Caregiving Organizations

Service providers from three caregiving organizations across Canada described their approaches to delivering education, information, and support to friend and family caregivers. While their programs were open to caregivers of all ages, service providers reported that most of the individuals they supported were over the age of 18. One organization noted an informal age requirement of 18 was intended, due to its programming being primarily designed for adults.

Caregivers accessing support from these organizations represented a wide range of socio-economic backgrounds including variation in age, culture, race, and income. Their caregiving roles also varied with their most commonly supported demographic consisting of parents caring for adult children, caregivers supporting family members from a distance, and those belonging to the sandwich generation (balancing responsibilities for their children and aging parents simultaneously).

#### 3.1.1. Offering Supports and Programming

All service providers provided a support line, described as a “warm line,” allowing caregivers to speak directly with staff rather than with an automated system:

*“They’re not just going to get a voicemail message, and if perchance they did, we would call them back right away.”*

(NS)

Some service providers also offer caregiving coaching programs and peer support groups designed to help caregivers navigate their roles. Participants also described efforts to expand their educational resources and spread awareness. Service providers from one organization stood out as leaders in supporting caregivers, by offering educational and learning resources through webinars, newsletters, websites and blogs. Participants from this organization reported facilitating a unique program for healthcare providers that used social prescribing (the holistic practice of healthcare that connects individuals to non-medical community resources) to facilitate the early identification of family caregivers, increasing overall referrals to their organization. This program also shares resources such as webinars, one-pagers, flipbooks, and E-news directly to an email list of healthcare providers.

Community partnerships were consistently identified as central to service delivery. Some service providers from organizations formed partnerships with other service providers, youth-serving groups, and schools to strengthen awareness and expand access to caregiving resources:

*“There’s also mental health workers. Sometimes there are RCMP officers, lawyers and organizations such as the [name of organization providing sexual health services]. So it’s all different people from the community that could potentially be people to refer young caregivers to us.”*

(NS)

Service providers emphasized the value of employing staff members with firsthand caregiving experience, either as current or past caregivers. Their lived experiences informs the organization’s mandates, allowing them to improve care and provide better service to clients:

*“The fact that we have caregivers on our team brings that perspective into the organization and into the conversation. And it allows us to see things in a different way for sure… I think it was a game changer.”*

(BC)

However, some service providers acknowledged that their staff were predominantly over the age of 30, which could create some barriers in engaging young caregivers, who may find it difficult to relate to older staff. Service providers emphasized the importance of including young caregivers on their team to bridge this gap.

#### 3.1.2. Accessing Caregiving Services

Service providers described differences in their intake processes and eligibility criteria for accessing their programs. Some reported having no formal intake process, while others required caregivers to complete structured phone assessments or meet additional criteria to self-identify as a caregiver. One service provider shared that their organization took pride in its accessibility, offering multiple entry points for support through its website, call line, and webinar events.

Many service providers across the organizations noted a common disconnect between the initial reasons individuals reached out for support and their actual needs:

*“We track reasons for calling and often the reason for calling doesn’t match what the real need is and there’s a disconnect. Certainly, during the pandemic one of the most interesting and pervasive concerns was financial. And when new money became available in the public promotional realm, the government was handing out money. There was thinking that there was extra money for people to care at home and there in fact wasn’t.”*

(BC)

Another individual reported that many callers to their organization misunderstood the services they offered, mistakenly assuming they were a continuing care organization that provided home care.

Participants reported that primary caregivers, who were typically adults, were the ones who most often approached the organization. Young caregivers rarely reached out independently and were often connected to support services through referrals from another adult caregiver or care recipient:

*“Anyone under the age of 18 would go through our regular intake and all the questionnaires we have right now. We haven’t had a lot of them, we’ve had some. The ones I’m aware of have come through a different care recipient or caregiver that we are supporting. They said ‘oh my grandson or my son, [whoever], would like some assistance as well’. But I don’t think we’ve had very many directly contact us.”*

(A)

### 3.2. Barriers and Challenges to Supporting Young Caregivers

Service providers reported not having restrictive protocols to access their services as they sought to be inclusive and allow for self-identification. If caregivers under 18 sought support, they were processed through the regular intake procedure, which focused on empathetic listening and holistic assessments to match them to the right supports. One service provider reflected on their approach of connecting a young caregiver who had approached the organization during COVID-19 for support:

*“I had talked to her about trying to connect her with other people. They weren’t necessarily young caregivers now, but they had been when they started their [caregiving] journey. So like I don’t think we have any specific youth targeted kind of programs… What I suggested was connecting her with somebody else who sort of had done the same journey, and that was with a parent [in the program].”*

(NS)

This approach highlights a significant gap in tailored services for younger caregivers and the need for more specific supports that connect them with other young caregivers.

#### 3.2.1. Recognition of Young Caregiver Role

A central barrier identified by participants was the limited recognition and self identification of young caregiving roles:

*“People don’t even recognize the role as an adult, so I’m not sure, even recognizing it as a child. We’d need to have people that kind of facilitate it and are aware. So it’s a lot of PR to get out there… to make sure that they recognize that they actually are a young caregiver. I mean, I think kids downplay ‘I’m just helping my mother’ or ‘looking after my grandmother, or my sibling’, or whoever that may be.”*

(NS)

Even within families, many guardians did not see the younger members of their family as caregivers, unless it was explicitly prompted:

*“It was very interesting, because most people were saying, ‘oh, I never really thought about my grandchildren, where my children are being caregivers. They’re just my kids and my grandkids.’”*

(BC)

#### 3.2.2. Barriers to Accessing Support

Some young caregivers faced disadvantages when seeking support due to their lack of a trusted and responsible guardian. This created barriers that limited their ability to get help or made them hesitant to seek support:

*“… they are the only caregiver for the situation. Maybe they are caring for their grandma who has dementia who has no way to consent for them. Or they’re getting to a point of burning out and they don’t want the person to know that they’ve called for help.”*

(A)

*“And in the more extreme format is if the parent is maybe not capable in terms of their parenting resources, and the children are filling the gap. There’s a need to keep that kind of under the table secret, because then it could bring the attention of authorities as well.”*

(NS)

Many service providers recognized the need for targeted approaches to engage younger caregivers, since they often lacked knowledge or access to available resources. Since young caregivers were often less mobile than older caregivers, service providers emphasized the need to adapt their outreach strategies to “meet [young caregivers] where they are,” without conflicting with their school schedules:

*“Most of our support groups right now are during the day. So that would be tricky for young caregivers who might be in school, or university, or whatever, to get to those. Also travel, like being able to get kind of travel to things would be something.”*

(NS)

As a result, demand has grown for organizations to shift towards non-traditional support approaches, such as dedicated virtual platforms:

*“Statistically, they go online… Even though the number of young caregivers is growing, we don’t have a targeted approach. We don’t have a targeted program. We don’t have services that are unique. And then we realized, and we did that with intention. We realized before we begin to reach out in a different way, we need to construct the most realistic, effective, approach we possibly can to serve them. Because they are so different. So that has been a conscious decision, and we know that we would have to do things differently.”*

(BC)

#### 3.2.3. Limited Funding and Capacity

Funding constraints were identified as a key barrier to developing youth-centred supports. Current organizational research emphasized that generalized approaches had been effective for adult caregivers but were insufficient for reaching young caregivers:

*“What we’ve determined already, through our years of research and getting ready to serve caregivers is, right now, we can serve them minimally and not be specialized. But we’re not seeing them. So that tells us we’re not reaching them. So we would have to do a number of things in addition, because we can’t stop serving other caregivers in our generalized way, because it’s been successful.”*

(BC)

Service providers noted that funding could be directly translated into increasing the expertise of staff and providing them with the capacity to develop more appealing promotional materials tailored to younger caregivers:

*“We know that we have to promote differently. Number two, we have to create materials that are relevant to that group, and they are different. Number three, we need to dedicate more time and space to intentionally gain expertise. So when you hire somebody new, our data tells us it takes, on average, one year to have a knowledgeable, skilled trained staff in the caregiving space. You can’t go find them in many places. So we need to actually groom and train people to understand the young caregiver population that is different and separate from other demographic groups of caregivers.”*

(BC)

Young caregivers are at a vulnerable stage in their development, making it essential to approach them with caution and sensitivity. Given that they are not autonomous decision-makers, service providers emphasized that there is a greater risk when engaging with them. Funding for hiring skilled coordinators specifically dedicated to young caregivers was identified as a beneficial way to provide them with adequate care.

Service providers highlighted limitations in capacity and research as barriers in advancing their support for young caregivers. Some noted a lack of understanding of the needs of young caregivers and how they differed from the older demographic. Given the restrictions of their currently programming, many service providers felt they lacked the capacity to develop new approaches:

*“As an organization, we’re spread fairly thin. And because of that we’re trying to cover a lot of ground individually with as tight an organizational fit as possible to get the most out of our time spent, and you know, developing a new approach from the ground up is very labour intensive. It’s administratively intensive. It would be a really big undertaking right now.”*

(BC)

#### 3.2.4. Policy and Infrastructure

Service providers emphasized that expanding into the young caregiver space would require development of an entirely new infrastructure and implementation of revised policies and procedures. Engagement strategies would also need to be restructured to include collaboration from diverse sectors such as education, which would serve as a critical entry point for reaching younger caregivers.

The new division of service would require specialized skills and competencies, revised training for volunteers, the development of communication protocols for engaging parents, and enhanced privacy protection practices. Revising existing service models would also involve accounting for resources that allow young caregivers to engage in the program while accommodating their caregiving responsibilities and providing incentives to encourage participation. Many service providers expressed the need for more analysis and education to determine how to realign their services to better support this demographic:

*“The model we have now, which is support groups, is not necessarily the model that’s going to work for young caregivers, because when we look at the young caregivers organizations in Ontario, I don’t think they have support groups. I think they have events and activities that these youth can come to and kind of feel supported and do things together. It’s not necessarily this support group model that we’re using. So there would be a lot of education we’d want to do to kind of see what actually makes sense in that situation.”*

(NS)

Service providers acknowledged that expansion of programming would require more formal partnerships with child-centred organizations in each province. However, some noted that these organizations were often not well-developed, making it challenging to establish meaningful relationships and/or practical collaborations. Building these partnerships would take time and require extensive labour and administrative effort. Legal and consent-related challenges were also noted as a significant limiting factor:

*“There are barriers with organizations that we could approach to partner with around protection of privacy, consent and protection of individuals who would be considered vulnerable because they’re underage.”*

(BC)

One service provider highlighted barriers that may arise from guardian-based approaches to consent:

*“I know there are a lot of young people who are caring for a parent who are drug users or have a drug addiction, so those parents wouldn’t have the capacity to provide consent as well.”*

(A)

### 3.3. Navigating a Pandemic

Service providers described how the COVID-19 pandemic significantly intensified caregiver duties, particularly for younger individuals, as formal systems such as schools and home healthcare services were disrupted. They noted that the pandemic broadened many existing caregiving environments, with lockdowns and travel restrictions transforming many households into intergenerational care spaces. Family members who typically did not reside together found themselves living in the same household due to school closures, the shutdown of care facilities and disruptions to home healthcare support.

This dynamic with individuals of all ages uniting to provide care, strengthened family networks of care and solidified their connections. Service providers reported that many of these networks of care were sustained even after the height of the pandemic, leading them to reflect on how caregiving roles were shared and defined in novel ways:

*“So I think about the psychosocial practical things that happened because of COVID. We learned a lot about who can help but in different ways. So we probably need to segment the kinds of things that happen in networks of care and the needs that people support that are outside the traditional definition of ‘I am a caregiver’.”*

(BC)

#### 3.3.1. Young Caregivers During the Pandemic

The pandemic led to many younger individuals to assume a caregiver role for the first time. A service provider from one organization reported that individuals aged 14 to 34 represented their highest proportion of new caregivers:

*“So what happened during the pandemic is a lot of younger caregivers got thrown in that have never been primary caregivers before and for all kinds of reasons, right, like provincial home supports were not available and the whole previous system fell apart. So we have a huge proportion of new caregivers that tend to be younger. They tended to not be long term caregivers. They were the new caregivers and they were caring for fewer hours and shorter periods of time.”*

(BC)

With the transformation of networks of care to include family members of all ages, younger individuals in the home frequently assumed caregiving roles, frequently supporting care when older caregivers were occupied with other responsibilities:

*“School work is meant to be done at home but if Dad’s on an important call, or Mom’s on an important call, who’s taking care of the other siblings at that moment?… Are they a young caregiver? Well, they are. They’re probably wiping noses, changing pants, making dinners in addition to keeping them busy by playing and with emotional support. And so at that point in time, I think about it. Maybe that ended once everything opened up and everybody went back to normal, but for a period of time we had a group of kids who did more and had more responsibility in life in general than ever before.”*

(BC)

Other younger individuals in households were able to support tasks such as delivering groceries to family members who were unable to leave the house. However, despite being the most mobile they were often the last to have access to the COVID-19 vaccine.

*“So when we think about the younger generation they can drive, they can get groceries, and they can go. They’re not often counted or considered to be a young caregiver, but they’re part of the support network.”*

(BC)

Financial strain during COVID-19 was a significant concern for caregivers. Service providers reported that many caregivers anticipated government funding to be available to compensate for increased care responsibilities in the home and were negatively affected when funding was not available.

*“There was thinking that there was extra money for people to care at home and there in fact wasn’t. If you put financial concern at the top of the list, that has also been validated across Canada with some of our colleagues who deliver the same kind of services as us, and it continues to be a very, very important element of the caregiving landscape.”*

(BC)

This gap in financial assistance from the government was especially impactful for younger caregivers, as their economic well-being and employment stability were not well-established. Many had to forgo educational and career opportunities to fulfill their caregiving responsibilities, further exacerbating their financial strain.

*“In particular for the younger group, their economic well being, their status of employment, their security is not well established. They might have foregone university, foregone jobs, in order to care and hold space in COVID, so that financial piece is huge. And I think we want to pay most attention to the most vulnerable people in the landscape of care paired with potential economic insecurity. And that younger group is right in the front.”*

(BC)

#### 3.3.2. Organizations Facing Demand in a New World

The pandemic led to many younger individuals to assume a caregiver role for the first time. One service provider reported that individuals aged 14 to 34 represented their highest proportion of new caregivers.

Service providers reported a significant increase in calls and referrals to their organization during the pandemic because more caregivers sought support, after many services had been limited or cancelled.

Some reported facing internal challenges such as leadership changes and staff layoffs due to pandemic. Some positions, such as outreach coordinator roles, were no longer needed with the closure or limited access of partner organizations and schools. Established relationships with schools and other partners were significantly disrupted, but service providers reported informally re-establishing them after the pandemic.

*“We identified the schools as the main partner. So that relationship looked different because of the pandemic in terms of who the schools were letting in, whether or not [young caregivers] were going to school at different points in the pandemic, those kinds of things. So I think that probably influenced it in that way.”*

(NS)

Despite these challenges, all service providers identified some positive organizational changes during the pandemic. For instance, all the service providers found that moving their support and coaching programs online improved accessibility and increased potential to engage younger caregivers who typically felt more comfortable with the virtual format.

*“One thing I think that was beneficial that came out of the pandemic was our virtual support groups. I don’t think we were doing that before and I feel like the younger generation is more keen on online platforms, and the accessibility of not having to leave your home to engage in services that way.”*

(NS)

*“I can say that the program has expanded its reach mostly because of online availability. So that has made it more accessible to people rurally and to people who are more comfortable with technology.”*

(A)

One participant highlighted that the shift to online programming during the pandemic created a new entry point for caregivers to engage with their organization:

*“During the pandemic, we used a different online presence like Facebook Live… and we get a lot of the sign up through advertising, through our e-news, through collaborations. And so during the pandemic we increased our reach and had a lot more people coming in to view webinars or re-watch specific webinars that we’ve already had.”*

(BC)

### 3.4. A Journey and Vision Worth Supporting

Across organizations, service providers consistently described being the most interested in learning more and finding resources, despite being the least confident about caregiving roles.

*“Young caregivers are the ones who’ve [done] the most learning about ‘what do I have to plan for here? What am I doing? I don’t know what I’m doing,’ kind of thing.”*

(BC)

Service providers from one organization saw this as an opportunity to try a different approach and developed a group aimed at individuals who were new to caregiving. While it presently supports caregivers of all ages, they expressed plans to broaden its development to support younger demographics.

#### 3.4.1. What We Need to Address the Needs of Caregivers

Service providers identified several recurring areas where caregivers have been seen seeking support. One organization’s analysis of website traffic found that common support searches included the following percent breakdown:

*“Caregiver well-being is the highest at 23%. Healthcare navigation at 10%, emotions of caregiving at 7%, financial at 7%.”*

(BC)

When exploring other inputs such as information from healthcare providers and directly from caregivers, they reported key themes of interest for caregivers as:

*“Burnout, burden, how to cope, communication, self care, dealing with conflict in family relationships, communication with health care providers, advocating for mental health and communicating for the betterment of their care recipient.”*

(BC)

These findings illustrate the need for more resources and programs focused on coping strategies, mental health advocacy, and self-care practices.

One service provider further highlighted the need to find a model that serves youth better:

*“I would think the goal of our current model is so that people don’t feel alone… that they know there’s support and other caregivers out there. We may not have the vast resources established to support a lone caregiver kind of in the early stages.”*

(NS)

Government policies were also identified as limiting, with funding models and stipends that often excluded young caregivers and the care they provided:

*“One of my pet peeves with our current government system is that there are some funding models that are available to caregivers where they can hire people to assist, but they are not allowed to hire relatives. I think with young caregivers, we might formalize that a little bit more if they were kind of seen in that role.”*

(NS)

Service providers reported that young caregivers often balanced competing demands such as school and part-time jobs with their caregiving contributions. The need for formalization from the government was emphasized, to recognize and better alleviate the financial burden of young individuals in these roles:

*“You also have youth that have competing demands. They have to go to school, they have to have part time jobs to potentially pay for school if they’re going on to higher education. We don’t recognize that as the potentially paid role in the support. You can hire your neighbor to support grandma, but you can’t hire one of your older grandchildren to support… I don’t know if there’s recognition, I guess. I’m saying in terms of what some of the young caregivers are doing already within the family, that isn’t sort of recognized even by our government organizations. I guess somehow, to formalize that even more [would be helpful].”*

(NS)

#### 3.4.2. Strengthening Capacity to Support Young Caregivers

Service providers consistently emphasized the significance of forming partnerships and collaborating across sectors to create a more cohesive support network. Partnerships with schools, universities, and child-centered organizations was seen as a critical approach to help young caregivers self-identify and to connect them to support systems. Collaborating with sectors such as healthcare and social services could also better support young caregivers and avoid duplicating efforts.

One service provider shared their strategy of positioning their organization as a bridge between existing community support and services:

*“There are many of us in Canada who do what we do and we don’t want to duplicate efforts. So as a hub and an opportunity for specialization… we could dip into the resource pool, pull from it for application in our province and be an intermediary, a bridge or a partner, without investing as deeply in the development of the tools and resources. We could be testers or piloters.”*

(BC)

Rather than duplicating or reinventing existing programs, service providers from the organization saw value in leveraging existing infrastructure to map out new connections in the existing ecosystem. They expressed the potential in forming provincial-to-federal connections to create comprehensive caregiving hubs alongside established young caregiving organizations that they already had strong connections with.

Additionally, one service provider shared that their organization considered partnering with existing youth programs that already had capacity and skills:

*“We would need to be offering some value or added tools. Like here’s the program, could we do an add-on program in your afterschool care called ‘Young caregivers 101’ or whatever it is. So the approach would be about having the tools and resources that could be implemented at the local level where we serve and function as the provincial bridge.”*

(BC)

To better support young caregivers, service providers recognized the need to shift away from traditional support group models to more engaging and dynamic approaches:

*“Our support groups are where we go out into the various communities to be present, but we’d have to be present in another format for youth in order to engage them. So we’d have to be more on-site, probably to where they frequent being… or a dedicated platform to entice them because virtual is probably the way they’re gonna go.”*

(NS)

## 4. Discussion

This study advances Canadian and international scholarship on young caregivers by examining the perspectives of caregiving organizations across multiple provinces that are attempting, or aspiring, to support young caregivers within adult-oriented service systems. Building on our earlier Ontario-focused work, these findings illuminate how organizational readiness, structural constraints, and policy invisibility shape the uneven landscape of support for young caregivers across Canada. By foregrounding organizational voices, this study shifts the analytical lens from individual caregiving burden to the systems and infrastructures that enable or constrain support for young caregivers.

### 4.1. Persistent Invisibility Within Adult-Centred Systems

Across provinces, organizations demonstrated strong commitment to supporting caregivers broadly, yet young caregivers remained largely invisible within service mandates, intake processes, and program design. This mirrors earlier Canadian findings indicating that young caregivers are rarely identified unless attached to an adult caregiver already engaged with services [1,15]. The absence of age-appropriate programming was not due to lack of interest, but rather to structural constraints, limited funding, and uncertainty regarding how to ethically and effectively engage minors.

This invisibility is reinforced by cultural norms that frame caregiving as a familial duty rather than a role warranting formal recognition, particularly when performed by children and youth. As service providers noted, young caregivers themselves often downplay their responsibilities, echoing international literature documenting low self-identification among young carers [29,30]. Without intentional identification mechanisms embedded in schools, healthcare, and community settings, young caregivers remain dependent on adult gatekeepers for recognition and referral, many who may not be aware of the needs of young caregivers.

### 4.2. Structural and Policy Barriers to Youth-Focused Programming

Participants consistently identified funding models, consent requirements, and risk management concerns as major barriers to developing youth-focused support services. These challenges align with international evidence showing that services for young caregivers require distinct infrastructure, safeguarding protocols, and staff competencies that differ from adult caregiver programs [31,32]. In Canada, where young caregivers lack formal recognition in federal or provincial policy, organizations are left to navigate these complexities without clear guidance or sustainable funding leading to barriers and inefficient resource allocation.

Unlike the UK, where young carers are legally recognized and entitled to assessments under the Care Act, Canadian organizations operate within fragmented systems that prioritize crisis response over prevention [12,29]. The findings suggest that without policy recognition and dedicated funding streams, organizations are forced to make strategic decisions that prioritize existing adult services at the expense of developing youth-specific supports.

### 4.3. COVID-19 as a Stress Test and Catalyst for Change

The COVID-19 pandemic functioned as both a stress test and a catalyst within the caregiving landscape. Consistent with prior research, organizations reported increased caregiving intensity, expanded family care networks, and heightened demand for support during periods of lockdown and service disruption [18,19]. For young caregivers, school closures and reduced formal supports eliminated key sources of respite, exacerbating stress and isolation.

At the same time, the pandemic accelerated innovation, seen through the rapid shift to virtual programming. Online supports were described as more accessible for younger caregivers, rural families, and those constrained by caregiving schedules. This finding aligns with emerging international evidence that digital and hybrid models may lower access barriers for young caregivers when implemented thoughtfully [32]. However, virtual delivery alone is insufficient; it must be paired with intentional outreach, youth-centred design, and safeguards to ensure confidentiality and trust.

### 4.4. Implications for Practice, Policy, and Research

Taken together, these findings highlight the need for a paradigm shift in how young caregivers are recognized and supported in Canada. From a practice perspective, caregiving organizations require evidence-informed models tailored to youth developmental stages, caregiving intensity, and family contexts. Tools such as early identification screeners (e.g., the Carers’ Alert Thermometer) and school-based referral pathways offer promising starting points [33].

From a policy perspective, formal recognition of young caregivers within federal and provincial frameworks is foundational. The development of a national strategy on young caregivers would provide a critical framework for aligning funding, service delivery and sustainability across Canada. Without it, funding remains precarious, service eligibility inconsistent, and organizational efforts fragmented. Cross-sector integration, particularly between education, healthcare, and community organizations is essential to move beyond reactive, adult-centric models and towards models that integrate elements of prevention and early intervention.

Future research should prioritize participatory and longitudinal designs that engage young caregivers directly, capturing how caregiving trajectories evolve over time and intersect with education, employment, and health outcomes [7,16,34]. Evaluations of virtual and hybrid interventions are also needed to determine what works, for whom, and under what conditions.

### 4.5. Strengths and Limitations

A key strength of this study is its multi-provincial scope, which extends the evidence base beyond Ontario and captures organizational perspectives across regions with varying levels of infrastructure, policy recognition, and service maturity for young caregivers. By including organizations from provinces with less-established young caregiver programming, this study provides a more realistic picture of Canada’s uneven caregiving landscape and highlights systemic issues that transcend provincial contexts. An additional strength lies in the study’s focus on organizational and service-provider perspectives, an underexplored yet critical vantage point in young caregiver research. While much of the existing literature centres on individual experiences of young caregivers, this study illuminates the structural, operational, and policy-level constraints that shape what support is possible in practice. This systems-level lens strengthens the study’s contribution to implementation, policy, and health services research. The diversity of professional roles represented among participants (including program coordinators, directors, outreach workers, educators, navigators, and social prescribing staff) further enhances the credibility and transferability of the findings. Capturing perspectives across organizational hierarchies and functions allowed for a more nuanced understanding of both strategic decision-making and frontline service delivery challenges. Moreover, the use of group interviews facilitated dialogue and collective reflection among participants, generating rich data about shared challenges, tensions, and aspirations within caregiving organizations.

Several limitations should also be acknowledged. The sample size was modest relative to the diversity and breadth of caregiving contexts across Canada, and participants largely reported being made aware of the study through organizations already engaged in, or interested in, supporting young caregivers. As such, perspectives from organizations with little awareness of young caregiving, or those not yet considering youth-focused programming, may be underrepresented. Data collection occurred in early 2023, which may raise questions about temporal relevance. However, given the slow pace of policy development, limited growth in targeted young caregiver programming, and continued lack of formal recognition in Canada, the barriers and challenges identified remain highly relevant and largely unresolved. Finally, while this study foregrounds organizational perspectives, it does not include the voices of young caregivers themselves, who may hold different views on service accessibility, relevance, and trust. This limitation underscores the importance of complementary research that directly engages young caregivers using participatory and youth-centred methodologies to more fully inform service design and policy development.

## 5. Conclusions

This study highlights the significant disconnect between the scale of young caregiving in Canada and the capacity of existing service systems to respond effectively. While caregiving organizations demonstrate a strong willingness to support young caregivers, their efforts remain constrained by adult-oriented mandates, limited and unstable funding, policy invisibility, and insufficient infrastructure. As a result, young caregivers continue to rely on indirect referrals, informal supports, or crisis-driven interventions rather than timely, preventative care.

Strengthening support for young caregivers requires coordinated action across policy, practice, and research. Early identification, youth-centred program design, virtual and school-based delivery models, and cross-sector partnerships are identified as critical priorities. Equally important is the meaningful involvement of young caregivers in co-designing services and policies that reflect their lived realities.

Investing in young caregivers is not only a matter of equity, but a strategic imperative for sustaining Canada’s caregiving and healthcare systems. By moving beyond fragmented, adult-centric approaches toward integrated and preventative models, Canada has the opportunity to better support young caregivers’ well-being, educational trajectories, and long-term health while strengthening the resilience of families and communities nationwide. In the long-term such investments will reduce the burden on the healthcare system and reduce spending on costly crisis-responsive interventions.

## Data Availability

The raw data supporting the conclusions of this article will be made available by the authors on request.
